# N-terminal pro-brain natriuretic peptide as a biomarker for predicting coronary artery lesion of Kawasaki disease

**DOI:** 10.1038/s41598-020-62043-6

**Published:** 2020-03-20

**Authors:** Xiaolan Zheng, Yi Zhang, Lei Liu, Peng Yue, Chuan Wang, Kaiyu Zhou, Yimin Hua, Gang Wu, Yifei Li

**Affiliations:** 1 Department of Pediatrics, West China Second University Hospital, Sichuan University, Chengdu, Sichuan 610041 China; 2 Key Laboratory of Birth Defects and Related Diseases of Women and Children of MOE, West China Second University Hospital, Sichuan University, Chengdu, Sichuan 610041 China

**Keywords:** Predictive markers, Aneurysm

## Abstract

Coronary artery lesion (CAL) caused by Kawasaki disease (KD) is currently the most common acquired heart disease in children in many countries. Nevertheless, there is no single useful marker existing for predicting CAL of KD. Recently, many reports have noted that N-terminal pro-brain natriuretic peptide (NT-proBNP) can be utilized as a biomarker to predict CAL. Thus, we perform a meta-analysis to ascertain the diagnostic value of NT-proBNP in detecting CAL of KD in the acute phase. PubMed, the Cochrane Central Register of Controlled Trials, EMBASE, and China National Knowledge Infrastructure were searched to detect relevant publications. Finally, eight eligible studies were included. The overall diagnostic sensitivity and specificity were 0.84 (95% confidence interval [CI]: 0.78–0.89) and 0.71 (95% CI: 0.68–0.75), respectively. The area under the summary receiver operating characteristic curves value (SROC) curve was 0.8582 ± 0.0531. Moreover, the overall sensitivity and specificity across five studies adopted the threshold of approximately 900 ng/L were 0.82 (95% CI: 0.73–0.89) and 0.72 (95% CI: 0.68–0.76), respectively. SROC was 0.8868 ± 0.0486. This meta-analysis would be the first one to describe the role of NT-proBNP in detecting CAL of KD. We register this study with PROSPERO (CRD42019130083).

## Introduction

Kawasaki disease (KD) is a systemic, self-limited vasculitis. However, its etiology is still unknown. The major adverse impacts of this disease are mainly dependent on the occurrence of coronary artery lesions (CALs) among children paitents^1^. At present, KD ranks the top prevalence in pediatric acquired heart diseases around the world, including developed and rapidly industrializing countries^2–4^. Intravenous immunoglobulin (IVIG) has been identified to help reduce the prevalence of CAL to about 4%^5^. As stratified initial treatment in patients with high predictive risk could reduce this risk, lots of studies have focused on exploring potential biomarkers to diagnosis KD or predict the rate of CAL in KD patients, for example, erythrocyte sedimentation rate (ESR) and C reactive protein (CRP). But the predictive values of recognizing biomarkers are partly limited^6^.

As a biomarker for the diagnosis and monitoring disease progression in heart failure, serum N-terminal pro-brain natriuretic peptide (NT-proBNP) has been globally endorsed in clinical guidelines^7,8^. Moreover, NT-proBNP has been considered as a potential diagnostic biomarker for KD^9,10^. Since Kaneko *et al*.^11^ first reported NT-proBNP levels may associate with the development of CAL in KD, and it can be considered as a valuable biomarker to predict the risk of CAL in acute KD before initial IVIG treatment. Although a series of studies^12–14^ on the diagnostic value of NT-proBNP to detect CAL of KD have been carried out in recent years, but they could not achieve a convincing result. Thus, we carried out this meta-analysis to ascertain the diagnostic accuracy of NT-proBNP in detecting CAL of KD in the acute period before initial IVIG treatment.

## Results

### Search results

**Figure 1:**
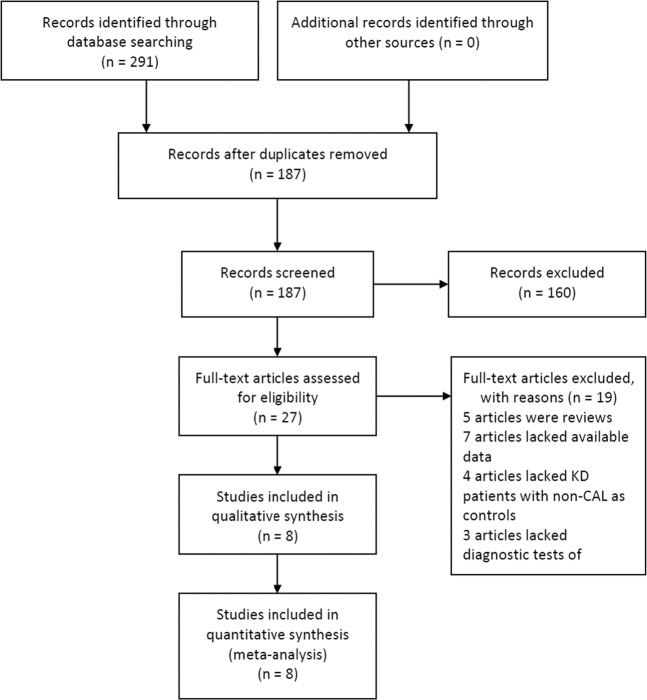
Flow diagram of the study selection.

A total of 291 potentially relevant reports were retrieved. Among them, 27 papers were picked up by review their titles and abstracts with great topic interests. Then 5 studies were excluded due to the following reasons, including inappropriate research design without a diagnostic test (n = 8), lacking available data to generate a 2 × 2 table (n = 7), and failed to provide control data (n = 4). Finally, 8 studies^11,14–20^ with 197 CAL patients and 664 non-CAL patients in acute KD were included in this analysis. The flow chart of the study selection was illustrated in Fig. 1. Among the enrolled studies, the study design of four were retrospective trials, and the remaining four articles were prospective trials. The basic characteristics of all enrolled studies were presented in Table 1.

**Table 1:** Characteristics of studies in meta-analysis.

No.	First author	Year	Countries	Design	Diagnostic criteria	Sample size of non-CAL/ CAL	Mean age of non-CAL/ CAL (month)	Mean NT-proBNP levels in patients with non-CAL (pg/ml)	Mean NT-proBNP levels in patients with CAL (pg/ml)	cut-off (pg/ml)	AUC	sensitivity	specificity
1	Kaneko K	2011	Japan	prospective	JKDRC	37/6	26.4/21.6	1,073	2,611	1,000	0.788	0.83	0.68
2	Qiu HX	2012	China	retrospective	AHA	77/25	21.5/13.3	865	1,726	827	0.803	0.84	0.70
3	Yoshimura K	2013	Japan	prospective	JKDRC	61/19	24.0/13.2	450	2,590	1,300	0.932	0.94	0.83
4	Chen YL	2014	China	prospective	CMA	26/9	24.0/20.4	590	1,023	900	0.77	0.78	0.73
5	Huiling L	2015	China	prospective	JKDRC	73/33	31.2/32.4	830	2,775	950	N/R	0.88	0.90
6	Lee HY	2016	Korea	retrospective	AHA	257/30	28.8/39.1	1,088	2,744	853	0.739	0.73	0.68
7	Fan JH	2018	China	retrospective	JKDRC	47/52	N/R	650	2,607	565	0.75	0.88	0.67
8	Jung JY	2019	Korea	retrospective	AHA	86/23	38.5/32.0	396	824	515	0.749	0.78	0.62

CAL = coronary artery lesion, NT-proBNP=N-terminal pro-brain natriuretic peptide, AUC = area under the curve, JKDRC = Japan Kawasaki Disease Research Committee, CMA = Chinese Medical Association, N/R = not report.

#### Study quality

We assessed the quality assessment of the included reports following the questions in the Quality Assessment of Diagnostic Accuracy Studies (QUADAS) list, and the results were illustrated in Table 2. Besides, all the eligible reports did not indicate follow-up time for CAL, which may be transient or permanent, which may increase bias the disease bias.

**Table 2:** QUADAS criteria of included studies.

No.	Spectrum composition	Selection criteria	Referencest-andard	Disease progression bias	Partial verification	Differential verification	Incor-poration bias	Index test execution	Reference standard execution	Test review bias	Reference standard review bias	Clinical review bias	Uninterruptible test results	With-drawals
1	?	+	+	?	+	+	+	+	+	+	+	+	+	?
2	?	+	+	?	+	+	+	+	+	?	+	+	?	+
3	+	+	+	?	+	+	+	+	+	+	+	+	+	?
4	+	+	+	?	+	+	+	+	+	+	+	+	+	?
5	+	+	+	?	+	+	+	+	+	?	+	+	+	?
6	+	+	+	?	+	+	+	+	+	?	+	+	+	+
7	?	+	+	?	+	+	+	+	+	?	+	?	+	+
8	+	+	+	?	+	+	+	+	+	+	+	+	+	+

#### Diagnostic accuracy of NT-proBNP in detecting KD with CAL

**Figure 2:**
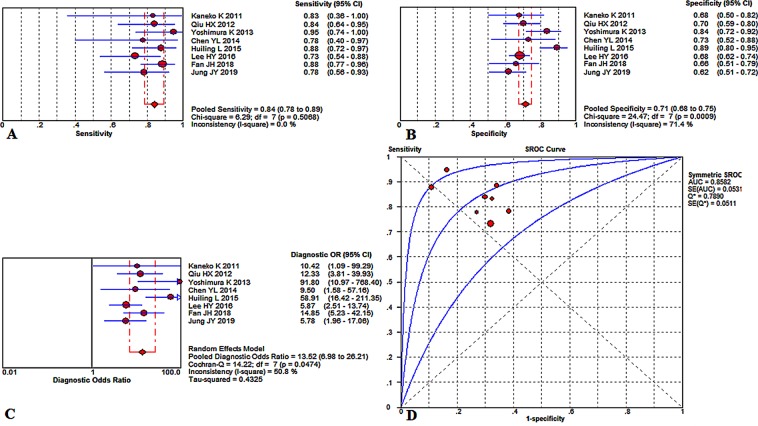
Overall performance of NT-proBNP detection for the diagnosis of KD with CAL in the acute phase of KD. (**A**) Pooled sensitivity. (**B**) Pooled specificity. (**C**) Overall DOR. (**D**) The SROCs for all datasets.

**Figure 3:**
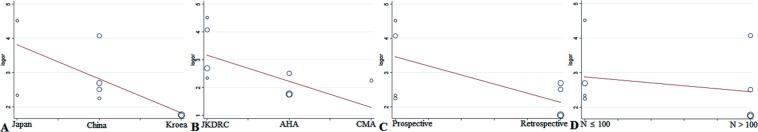
The meta-regression of the enrolled studies. (**A**) For the study country. (**B**) For the diagnostic criteria of CAL. (**C**) For the study design. (**D**) For the total sample size.

**Figure 4:**
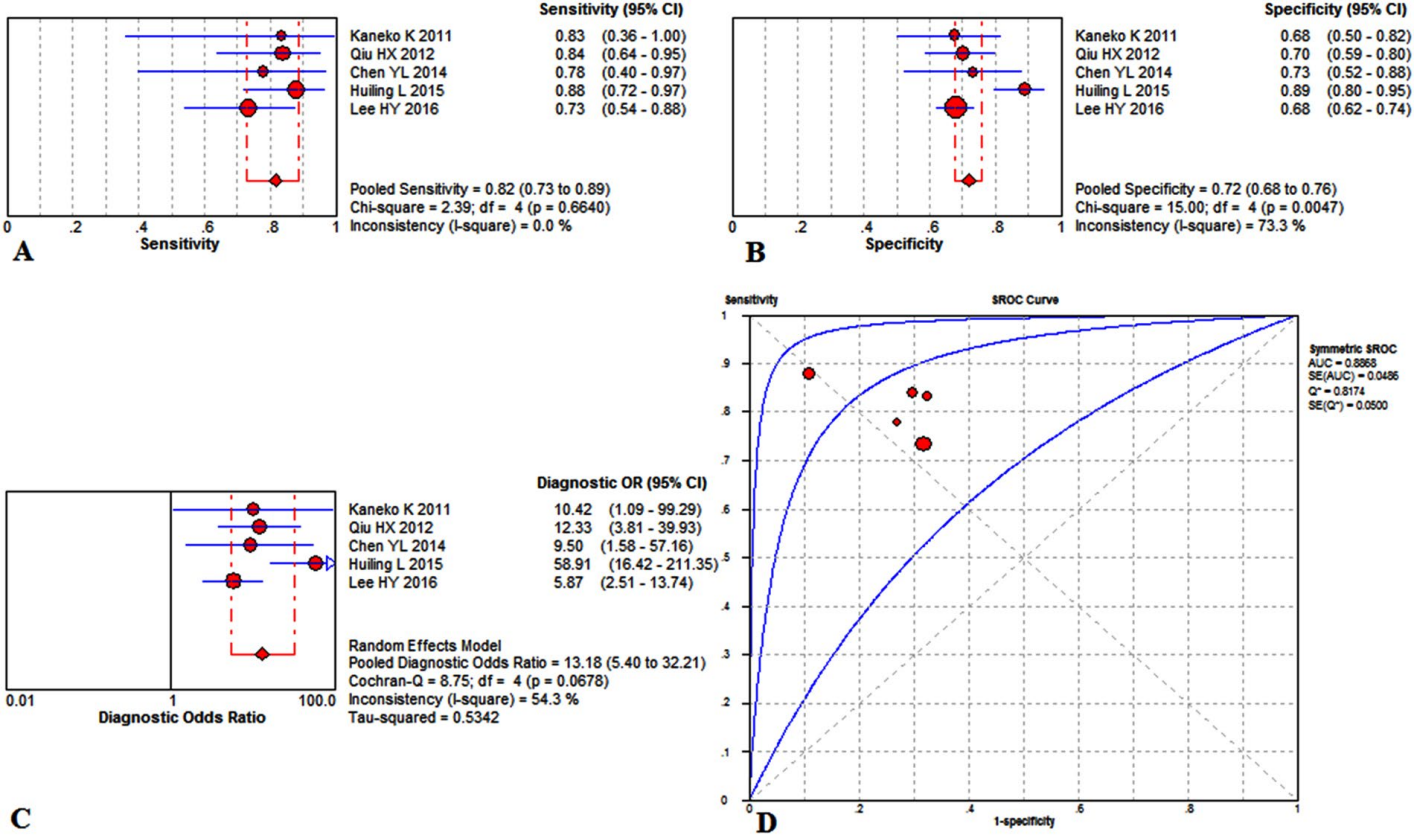
Performance of NT-proBNP (thresholds ≈ 900 ng/L) detection for the diagnosis of KD with CAL in the acute phase of KD. (**A**) Pooled sensitivity. (**B**) Pooled specificity. (**C**) Overall DOR. (**D**) The SROCs for all datasets.

Figure 2 summarizes the overall diagnostic value to predict KD with CAL by the level of NT-proBNP, demonstrating an overall sensitivity of 0.84 (95%CI, 0.78 to 0.89) with no significant heterogeneity (P = 0.5068, x^2^ = 6.29, I^2^ = 0.0%) (Fig. 2A), a specificity of 0.71 (95%CI, 0.68–0.75) combining a noticeable heterogeneity (P = 0.0009, x^2^ = 24.47, I^2^ = 71.4%) (Fig. 2B), and a diagnostic odds ratio (DOR) of 13.52 (95% CI, 6.98–26.21) with significant heterogeneity (P = 0.0474, Cochran-Q = 14.22, I^2^ = 50.8%) (Fig. 2C). Its area under the summary receiver operating characteristic curves (SROC) value (AUC) was counting as 0.8582 ± 0.0531 (Fig. 2D). The potential threshold effect is failed to be detected. Meta-regression has been used to measure the factors in inducing the heterogeneities, such as population nationalities, diagnostic criteria of CAL, study design, and total sample size. The meta-regression revealed the population nationalities made a significant contribution to the homogeneity, P = 0.037, t = −2.66, 95%CI (0.15, 0.92) (Fig. 3A). Besides, the diagnostic criteria of CAL was not a dramatic impact factor, P = 0.080, t = −2.11, 95%CI (0.13, 1.16) (Fig. 3B). Meanwhile, the meta-regression also did not detect the study design has a dramatic impact on the homogeneity of the enrolled studies too, P = 0.056, t = −2.37, 95%CI (0.07, 1.05) (Fig. 3C). Also, the total sample size is not a dramatic impact factor, P = 0.581, t = −0.58, 95%CI (0.11, 3.93) (Fig. 3D). Therefore, the differences among the study countries were the source of existing heterogeneity. Additionally, it’s interesting that we found there were five studies^11,15–18^ all had thresholds at approximately 900 ng/L. Therefore, we evaluated those studies and found the summary sensitivity was 0.82 (95%CI, 0.73–0.89), and the pooled estimation showed no significant heterogeneity (P = 0.6640, x^2^ = 2.39, I^2^ = 0.0%) (Fig. 4A). Meanwhile, the summary specificity was 0.72 (95%CI, 0.68–0.76), and the pooled estimation showed significant heterogeneity (P = 0.0047, x^2^ = 15, I^2^ = 73.3%) (Fig. 4B). The pooled DOR was 13.18 (95% CI, 5.40–32.21) with significant heterogeneity (P = 0.0678, Cochran-Q = 8.75, I^2^ = 54.3%) (Fig. 4C). The calculated AUC value was 0.8868 ± 0.0486 (Fig. 4D).

#### Sensitivity analysis and subgroup analysis

**Figure 5:**
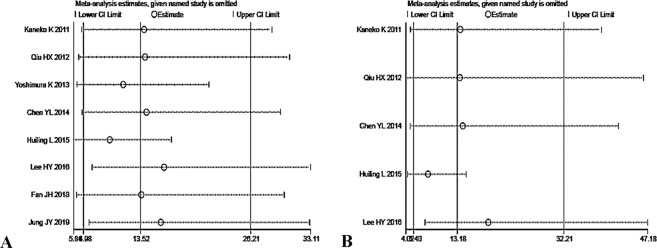
Sensitivity analysis of the individual trials on the results. (**A**) For the result of overall NT-proBNP, (**B**) For the result of NT-proBNP (thresholds ≈ 900 ng/L).

We systematically and qualitatively analyzed the sensitivity of the included studies to determine the influence of individual trials on the results of overall NT-proBNP and NT-proBNP (thresholds ≈ 900 ng/L), using STATA 15.1 for meta-analysis random-effects estimates. Finally, none of the studies was detected to incur undue weight in the analysis (Fig. 5). Besides, Meta-Disc 1.4 was utilized to detect whether there is any threshold effect in studies, and the result suggested that the Spearman correlation coefficient was −0.286 and P = 0.955, which was indicating no threshold effect related to heterogeneity existed. After then, we conducted two subgroup analyses by the study design and the total sample size. The pooled DOR of the prospective group was 32.52 (95% CI, 14.12–74.89) with low heterogeneity (P = 0.2094, Cochran-Q = 4.53, I^2^ = 33.8%), and the calculated AUC value was 0.9065 ± 0.0577. Besides, the pooled DOR of the retrospective group was 8.41 (95% CI, 5.19–13.62) with no significant heterogeneity (P = 0.4407, Cochran-Q = 2.70, I^2^ = 0.0%), and the calculated AUC value was 0.7357 ± 0.0692. Meanwhile, the pooled DOR of the total sample size (n ≤ 100) group was 18.37 (95% CI, 8.76–38.54) with no significant heterogeneity (P = 0.3729, Cochran-Q = 3.12, I^2^ = 4%), and the calculated AUC value was 0.8064 ± 0.1173. Additionally, the pooled DOR of the total sample size (n > 100) group was 11.73 (95% CI, 4.36–31.54) with moderate heterogeneity (P = 0.0184, Cochran-Q = 10.02, I^2^ = 70.1%), and the calculated AUC value was 0.8976 ± 0.0522. These results were shown in Table 3.

**Table 3:** Subgroup analysis results of included studies.

	Sensitivity (95% CI)	Specificity(95% CI)	DOR(95% CI)	SROC(AUC ± SE)
Total	0.84(0.78–0.89)	0.71(0.68–0.75)	13.52(6.98–26.21)	0.8582 ± 0.0531
P/I^2^	0.5068/0.0%	0.0009/71.4%	0.0474/50.8%	—
**Study design**				
Prospective	0.88(0.78–0.95)	0.81(0.75–0.86)	32.52(14.12–74.89)	0.9065 ± 0.0577
P/I^2^	0.6029/0.0%	0.0366/64.7%	0.2094/33.8%	—
Retrospective	0.82(0.75–0.88)	0.67(0.63–0.71)	8.41(5.19–13.62)	0.7357 ± 0.0692
P/I^2^	0.3498/8.7%	0.6583/0.0%	0.4407/0.0%	—
**Total sample size**				
N ≤ 100	0.88(0.80–0.94)	0.74(0.66–0.80)	18.37(8.76–38.54)	0.8064 ± 0.1173
P/I^2^	0.6031/0.0%	0.1400/45.2%	0.3729/4.0%	—
N > 100	0.81(0.73–0.88)	0.70(0.66–0.74)	11.73(4.36–31.54)	0.8976 ± 0.0522
P/I^2^	0.4861/0.0%	0.0004/83.6%	0.0184/70.1%	—

CI = confidence interval, DOR = diagnostic odds ratio, SROC = summary receiver operating characteristic curves value, AUC = area under the curve, SE = standard error.

#### Publication bias

**Figure 6:**
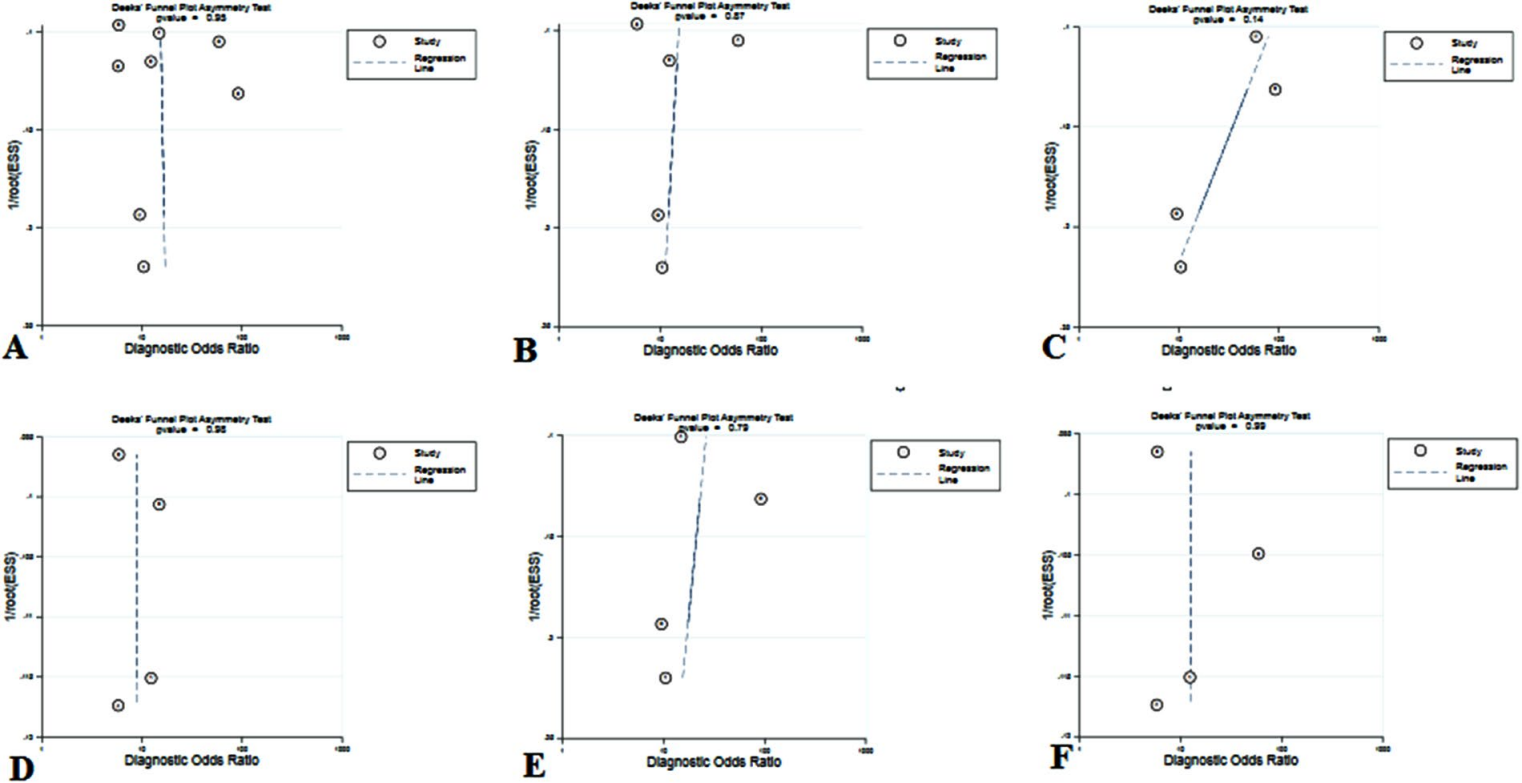
Funnel plots for the assessment of potential publication bias. The funnel graphs plot the Bsquare root of the effective sample size (1/ESS1/2) against the DOR. Each circle represents each study in the meta-analysis. Asymmetry of the circle distribution between regression lines indicates potential publication bias. (**A**) Total pooled result, (**B**) Thresholds ≈ 900 ng/L pooled result, (**C**) Prospective pooled result, (**D**) Retrospective pooled result, (**E**) Total sample size (n ≤ 100) pooled result, (**F**) Total sample size (n > 100) pooled result.

We used funnel plots and the Deeks’ test to assess publication bias in the included studies. Each dot plots in these graphs represented a study. The distance between each dot and the vertical line indicated bias in each study. Symmetric distribution indicated no publication bias existed. Funnel plots in Fig. 6A–F present a degree of symmetry, suggesting that there is no potential for publication bias among the included articles.

## Discussion

The diagnostic accuracy of NT-proBNP in detecting KD with CAL in the acute phase was systematically evaluated in our study. Finally, we found that NT-proBNP can be a valuable biomarker for predicting CAL of KD. The previous meta-analysis^10^ showed that NT-proBNP level was mildly higher than in KD patients of the acute phase compared with the febrile control patients. In our study, we found that the mean NT-proBNP level in KD patients with CAL was much higher than in KD patients without CAL (approximately 2500 ng/L vs. 800 ng/L) (Table 1). However, the precise mechanism of elevated NT-proBNP in KD patients is still unclear^21^. Nevertheless, factors known to affect the NT-proBNP levels, such as cardiac function^22^ or inflammatory cytokines^23^, were not evaluated in all the included studies, which could cause biased results. Therefore, further well-designed studies are needed to evaluate the value of NT-proBNP in predicting CAL of KD.

The present study suggested that the overall diagnostic sensitivity and specificity of NT-proBNP for diagnosis KD with CAL in the acute phase were 0.84 and 0.71, AUC of SROC was 0.8582. Then, we evaluated five studies, which all had thresholds at approximately 900 ng/L. Finally, we found the overall diagnostic sensitivity and specificity of NT-proBNP (threshold ≈ 900 ng/L) for diagnosis KD with CAL were 0.82 and 0.72, AUC of SROC was 0.8868, which was slightly higher than the overall diagnostic accuracy of NT-proBNP. In general, those found suggested that NT-proBNP can be used as a biomarker for detecting CAL of KD. Additionally, the diagnostic accuracy in the threshold of about 900 ng/L shows a little higher than the overall diagnostic accuracy, which still only indicates that NT-proBNP has diagnostic value, but cannot indicate that 900 ng/L is the recommended threshold. The specific threshold range needs to be determined by more well-designed clinical studies with larger sample size. Besides, the meta-regression showed that the differences among the study countries were the source of heterogeneity.

Furthermore, we conducted two subgroups analysis by the study design and the total sample size, and these results suggested that both the type of research and the total sample size might be the sources of heterogeneity. Besides, the calculated AUC of value was 0.9065 ± 0.0577 for the prospective group and 0.8976 ± 0.0522 for the total sample size (n > 100) group, which showed prospective studies with large sample size (n > 100) were superior designed studies to evaluate the value of NT-proBNP in predicting CAL of KD.

Although CAL in KD may be transient or permanent^24^, all the included studies did not provide follow-up time for CAL, which may bias the diagnostic value of NT-proBNP. Further rigorous studies, with unified inclusion and exclusion criteria and a consecutive enrolment design, needed to evaluate the diagnostic value of NT-proBNP in KD with CAL diagnosis.

Our meta-analysis has several limitations. First, the number of included studies was small (n = 8), and all of them were conducted in Asian populations, which means these results may not generalize to other areas. Second, no included articles combine NT-proBNP with other laboratory tests, such as ESR, CRP, to identify the diagnostic accuracy of KD with CAL, which could work as a better method for detection. Third, all of the follow-up time of the included articles were unclear, which may lead to the deviation of CAL diagnosis, which could further affect the accuracy of NT-proBNP in predicting CAL of KD.

In conclusion, despite these limitations, this is the first meta-analysis that showed NT-proBNP could be used as a biomarker for detecting CAL of KD. Besides, further well-designed studies with a large sample size are needed to strictly evaluate the value of NT-proBNP in predicting CAL of KD.

## Materials and Methods

### Study protocol and ethics statement

We performed this analysis following a predetermined protocol according to the recommendations of Deeks^25^. The data collection and reporting were by the Preferred Reporting Items for Systematic Reviews and Meta-Analyses (PRISMA) Statement^26^ (S Table 1). Due to it is a systematic literature study, ethical approval was not necessary. The protocol for this analysis was registered with PROSPERO (CRD42019130083).

### Search strategy

PubMed, the Cochrane Central Register of Controlled Trials (CENTRAL), EMBASE, and China National Knowledge Infrastructure (CNKI) through Mar 13th, 2019 were searched to detect relevant studies. Search terms of PubMed database were (Mucocutaneous Lymph Node Syndrome[MeSH Terms] OR Kawasaki disease OR Kawasaki syndrome) AND (pro-brain natriuretic peptide[MeSH Terms] OR NT-proBNP OR N-terminal pro-BNP OR NTproBNP OR NT-BNP). Search terms for the CENTRAL, EMBASE, and CNKI with the corresponding search results are listed in S Appendix 1. English and Chinese were the language limits for retrieval.

### Study selection

Articles were screened preliminarily by title and abstract after citations selected by the systematic search. And then, potentially relevant articles were retrieved by full text, while assessed for compliance to inclusion and exclusion criteria.

Inclusion criteria: (1) all cases must meet the KD diagnostic criteria; (2) randomized or non- randomized controlled, cohort studies, clinical trials evaluating NT-proBNP in blood samples; (3) contained the data that can calculate true positive (TP), false negative (FN), false positive (FP), and true negative (TN), such as specificity, sensitivity and sample size; (4) all studies had KD with non-CAL subjects as the control group; (5) The samples were taken from patients with acute KD before initial IVIG treatment.

Exclusion criteria: (1) reviews, editorials, abstracts, letters, expert opinions, conferences articles, or case reports without controls; (2) unable to construct 2 × 2 table; (3) duplicated publications.

### Data collection and assessment of study quality

The eligibility of studies was assessed by two investigators (Xiaolan Zheng, Yi Zhang) independently by the title and abstract. At the same time, the divergences and the quality of reports were determined by a third reviewer (Yifei Li) according to inclusion or exclusion criteria. The quality assessment of all included reports was evaluated by the two investigators (Xiaolan Zheng, Lei Liu) independently following the QUADAS list^27^. As well-conducted research might score lower in the absence of relevant parts of the methodology and results, the assessments were reported in descriptive form only. Finally, the data from which can calculate TP, FP, FN, and TN were extracted by two investigators (Xiaolan Zheng, Peng Yue).

### Evaluation indicators

We measured the following indicators of NT-proBNP: sensitivity, specificity, DOR, and SROC. Sensitivity represented the proportion of patients in KD patients with CAL, which were correctly identified by the positive results of NT-proBNP. Besides, specificity expressed the KD cases with non-CAL that were correctly identified by the negative results of NT-proBNP. Also, the DOR more reliably defined a summary of test performance, rather than merely pooling specificity and sensitivity in individual reports. DOR was an independent index with a range of 0 ~ infinity. The higher the DOR, the better the discrimination^28^. The SROC was plotted by combining sensitivity and specificity. Furthermore, AUC was calculated as a global measurement of test performance^29^, and the closer the AUC was to 1, the better the test performance would be.

### Publication bias

Funnel plots and the Deeks’ test were used to assess the publication bias. It indicated a potential publication bias when the asymmetric distribution of data dot in the funnel plot with a quantified result of P < 0.05^30^.

### Heterogeneity and meta-regression

The heterogeneity of pooling sensitivity and specificity were examined by the x^2^ test, while the heterogeneity of pooling DOR was examined by the Cochran Q test. The I^2^ test in every pooling analysis to quantitatively was also conducted to assess the proportion of total variation in the study. I^2^ value would range from 0 to 100%, with values of 25, 50, and 75%, respectively, as evidence of low, moderate, and high heterogeneity^31^. The threshold effect was suggested by a curvilinear shape in the SROCs. Furthermore, the meta-regression was carried out to detect the potential factors that would cause heterogeneity. All the possible factors were extracted from the baseline measurement and original testing procedures, which were included in meta-regression. The meta-regression can determine the correlation between the potential factors and the existing heterogeneity. The factor should have a dramatic impact on the homogeneity of the enrolled studies with a P-value < 0.05 when a significant difference was discovered.

### Sensitivity analysis

We performed the sensitivity analysis to determine the influence of individual studies on the results. Meta-Disc 1.4^32^ was used for detecting threshold effects in reports.

### Statistical analysis

Meta-Disc 1.4 was utilized to perform data analysis. Besides, STATA 15.1 (Stata Corporation, College Station, Texas, USA) was utilized to assess the publication bias and perform meta-regression analysis. Homogenous results utilized the random-effects model for statistical analysis, while the heterogeneous (I^2^ < 50%) results utilized a fixed-effects model, and the data were presented using a forest map.

## Supplementary information

Supplementary Information.

## Data Availability

The authors confirm that all the data based findings are fully available without restriction. All relevant data are included in the paper and references.
