# Supplementary material for: N-terminal pro-brain natriuretic peptide as a biomarker for predicting coronary artery lesion of Kawasaki disease
Source: Sci Rep. 2020 Mar 20;10:5130. doi: 10.1038/s41598-020-62043-6 (PMC7083930; doi:10.1038/s41598-020-62043-6)
Supplement: Supplementary file 1 — Supplementary Information. [file 41598_2020_62043_MOESM1_ESM.pdf]

N-terminal pro-brain natriuretic peptide as a biomarker for predicting coronary artery lesion of Kawasaki disease

Xiaolan Zheng[1,2], Yi Zhang [1,2], Lei Liu[1,2], Peng Yue[1,2], Chuan Wang [1,2], Kaiyu Zhou[1,2], Yimin Hua[1,2], Gang Wu[1,2] & Yifei Li[1,2]

S Table 1. PRISMA 2009 Checklist

Section/topic	#	Checklist item	Reported on page #
**TITLE**			
Title	1	N-terminal pro-brain natriuretic peptide as a biomarker for predicting coronary artery lesion of Kawasaki disease	1
ABSTRACT			
Structured summary	2	Coronary artery lesion (CAL) caused by Kawasaki disease (KD) is currently the most common acquired heart disease in children in many countries. However, there is no single useful marker existing for predicting CAL of KD. Recently, many reports have noted that N-terminal pro-brain natriuretic peptide (NT-proBNP) can be used as a marker to predict CAL. Thus, we perform this meta-analysis to ascertain the diagnostic value of NT-proBNP in detecting CAL of KD in the acute phase. We searched PubMed, the Cochrane Central Register of Controlled Trials, and China National Knowledge Infrastructure. Finally, eight eligible studies were included. The overall diagnostic sensitivity and specificity were 0.84 (95% confidence interval [CI]: 0.78 - 0.89) and 0.71 (95% CI: 0.68 - 0.75), respectively. The area under the summary receiver operating characteristic curves value (SROC) curve was 0.8582 ± 0.0531. Moreover, the overall sensitivity and specificity across five studies adopted the threshold of approximately 900 ng/L were 0.82 (95% CI: 0.73 - 0.89) and 0.72 (95% CI: 0.68 - 0.76), respectively. SROC was 0.8868 ± 0.0486. This is the first meta-analysis showed that NT-proBNP could be used as a biomarker for detecting CAL of KD, and the recommended threshold was 900 ng/L.	1
INTRODUCTION			
Rationale	3	Kawasaki disease (KD) is a self-limited systemic vasculitis of unknown cause, which is associated with the development of coronary artery lesions (CALs) occurring in children under five years old. KD is currently the most common cause of acquired heart disease in children in many countries, including developed and rapidly industrializing countries. Timely initiation of treatment with intravenous immunoglobulin (IVIG) has reduced the risk of CAL from 25% to about 4%. As stratified initial treatment for the patients with a higher predicted risk might reduce this risk, lots of studies have focused on exploring potential biomarkers to diagnosis KD or predict the risk of CAL in patients with KD, such as erythrocyte sedimentation rate (ESR) and C reactive protein (CRP), but there is still no useful single biomarker existing. As a biomarker to aid in the diagnosis of heart failure and to monitor disease progression, serum N-terminal pro-brain natriuretic peptide (NT-proBNP) has been globally endorsed in clinical guidelines7,8. Moreover, NT-proBNP has been recognized as a useful marker for the diagnosis of KD. Since Kaneko et al11 first reported NT-proBNP levels may associate with the development of CAL in KD, and it can be considered as a useful marker to predict the risk of CAL in acute KD before initial IVIG treatment, a series of studies12-14 on the diagnostic value of NT-proBNP in detecting CAL of KD have been carried out in recent years, but the results have not always been consistent.	1-2

Objectives	4	We perform this meta-analysis to ascertain the diagnostic accuracy of NT-proBNP in detecting CAL of KD in the acute phase before initial IVIG treatment.	2
METHODS			
Protocol and registration	5	This analysis was performed by a predetermined protocol following the recommendations of Deeks. The data collection and reporting accorded with the Preferred Reporting Items for Systematic Reviews and Meta-Analyses (PRISMA) Statement. The ethical approval was not necessary due to it is systematic literature research.	4-5
Eligibility criteria	6	Criteria for inclusion: (1) all patients of KD were confirmed by KD diagnosis criteria; (2) randomized controlled or non-randomized controlled, clinical trials, cohort studies evaluating NT-proBNP in blood samples; (3) contained the date which can calculate true positive (TP), false positive (FP), false negative (FN), and true negative (TN), such as sensitivity, specificity, and essential sample size; (4) all studies had KD with non-CAL subjects as the control group; (5) blood samples were obtained in the acute phase of KD before initial IVIG treatment.<br>Criteria for exclusion: (1) conferences articles, reviews, editorials, abstracts, letters, expert opinions, or case reports without controls; (2) no available data to construct a 2×2 table; (3) duplicated reports.	5
Information sources	7	We searched multiple databases including PubMed, EMBASE, the Cochrane Central Register of Controlled Trials, and China National Knowledge Infrastructure through Mar 13th, 2019 to identify relevant studies.	5
Search	8	Keyword search terms were ('mucocutaneous lymph node syndrome' OR 'Kawasaki disease' OR 'Kawasaki syndrome') AND ('pro-brain natriuretic peptide' OR 'NT-proBNP' OR 'N-terminal pro-BNP' OR 'NTproBNP' OR 'NT-BNP'). PubMed database was searched as follows: (Mucocutaneous Lymph Node Syndrome[MeSH Terms] OR Kawasaki disease OR Kawasaki syndrome) AND (pro-brain natriuretic peptide[MeSH Terms] OR NT-proBNP OR N-terminal pro-BNP OR NTproBNP OR NT-BNP).	5
Study selection	9	Reports were preliminarily screened by title and abstract and when initially selected by the systematic search. Potentially relevant studies were then retrieved by full manuscripts and assessed for compliance with inclusion and exclusion criteria.	5
Data collection process	10	Two investigators (Xiaolan Zheng, Yi Zhang) assessed the eligibility of studies by reading the title or abstract independently. A third reviewer (Yifei Li) determining the divergences according to inclusion or exclusion criteria, and the quality of reports. Two investigators (Xiaolan Zheng, Lei Liu) independently evaluated the quality assessment of all enrolled studies following the 14-item Quality Assessment of Diagnostic Accuracy Studies (QUADAS) list. As well-conducted research might score lower in the absence of relevant parts of the methodology and results, the assessments were only reported in descriptive forms. Finally, two investigators (Xiaolan Zheng, Peng Yue) extracted the date from which can calculate TP, FP, FN, and TN.	5
Data items	11	The data of TP, FP, FN, and TN, such as sensitivity, specificity, and essential sample size.	5
Risk of bias in individual studies	12	We used Stata statistical software (STATA, version 15.1) to obtain a quantitative analysis of all the publication bias according to funnel plots and the Deek's test. An asymmetric distribution of data points in the funnel plot with a quantified result of P<.05 indicated the presence of potential publication bias	6
Summary measures	13	The following indicators of different types of miRNAs were measured: sensitivity, specificity, diagnostic odds ratio (DOR), and area under the summary receiver operating characteristic curves value (SROC).	5-6
Synthesis of results	14	Sensitivity, specificity, diagnostic odds ratio (DOR), and area under the summary receiver operating characteristic curves value (SROC).	5-6

Section/topic	#	Checklist item	Reported on page #

Risk of bias across studies	15	We used Stata statistical software (STATA, version 15.1) to obtain a quantitative analysis of all the publication bias according to funnel plots and the Deeks' test. An asymmetric distribution of data points in the funnel plot with a quantified result of $P<.05$ indicated the presence of potential publication bias	6
Additional analyses	16	We carried out the meta-regression analysis using STATA 15.1 to detect where the potential factor for heterogeneity origin from. Sensitivity analysis was conducted for every study to determine the influence of individual trials on the results, using STATA 15.1 for meta-analysis fixed/random-effects estimates. Meta-Disc 1.4 was used to detect threshold effects in studies and conduct subgroup analysis.	6
RESULTS			
Study selection	17	Initially, 291 potentially relevant papers were retrieved by the search method aforementioned, of which 27 articles were considered to be interested after reading titles and abstracts. However, five articles were excluded by reading their complete articles due to article types, seven studies lacked available data to construct a 2×2 table, four articles lacked KD patients with non-CAL as controls, and three studies lacked diagnostic tests of interest. Finally, eight studies with 197 CAL patients and 664 non-CAL patients in acute KD were included in the meta-analysis. The flow diagram of the study selection was illustrated in Figure 1.	2
Study characteristics	18	Among the enrolled studies, the study design of four were retrospective trials, and the remaining four studies were prospective trials. Besides, the total sample size of four studies was larger ($n > 100$) compared the remaining four ($n \le 100$) The basic characteristics of all included studies were shown in Table 1.	2
Risk of bias within studies	19	Funnel plots were used to evaluate the publication bias of the included studies. Each dot plots in these plots represented a study. The distance between each dot and the vertical line indicated bias in each study. The absence of any asymmetric distribution suggested that there was no publication bias. Funnel plots in Figure 6A-F present a certain degree of symmetry, which suggested no potential for publication bias among the studies included in this meta-analysis.	3
Results of individual studies	20	A total of eight relevant studies were eligible to analyze pooled accuracy. The overall diagnostic measurement in detecting KD with CAL of NT-proBNP has been summarized in figure 2. The summary sensitivity was 0.84 (95%CI, 0.78 - 0.89), the summary specificity was 0.71 (95%CI, 0.68 - 0.75), and the pooled estimation showed noticeable heterogeneity (P=.0009, x2=24.47, I2=71.4%). The pooled diagnostic odds ratio (DOR) was 13.52 (95% CI, 6.98 - 26.21), SROC was 0.8582±0.0531. Additionally, it's interesting that we found there were five studies all had thresholds at approximately 900 ng/L. Therefore, we evaluated those studies and found the summary sensitivity was 0.82 (95%CI, 0.73 - 0.89), the summary specificity was 0.72 (95%CI, 0.68 to 0.76), and the pooled DOR was 13.18 (95% CI, 5.40 - 32.21). The calculated AUC value was 0.8868±0.0486.	2-3
Synthesis of results	21	The overall diagnostic measurement in detecting KD with CAL of NT-proBNP has been summarized in figure 2. The summary sensitivity was 0.84 (95%CI, 0.78 - 0.89), the summary specificity was 0.71 (95%CI, 0.68 - 0.75), and the pooled estimation showed noticeable heterogeneity (P=.0009, x2=24.47, I2=71.4%). The pooled diagnostic odds ratio (DOR) was 13.52 (95% CI, 6.98 - 26.21), SROC was 0.8582±0.0531. Additionally, it's interesting that we found there were five studies all had thresholds at approximately 900 ng/L. Therefore, we evaluated those studies and found the summary sensitivity was 0.82 (95%CI, 0.73 - 0.89), the summary specificity was 0.72 (95%CI, 0.68 to 0.76), and the pooled DOR was 13.18 (95% CI, 5.40 - 32.21). The calculated AUC value was 0.8868±0.0486.	2-3
Risk of bias across studies	22	Funnel plots were used to evaluate the publication bias of the included studies. Each dot plots in these plots represented a study. The distance between each dot and the vertical line indicated bias in each study. The absence of any asymmetric distribution suggested that there was no publication bias. Funnel plots in Figure 6A-F present a certain degree of symmetry, which suggested no potential for publication bias among the studies included in this meta-analysis.	3
Additional analysis	23	The meta-regression detected the differences among the study countries had a dramatic impact on the homogeneity of the enrolled studies, P=.037, t=-2.66, 95%CI (0.15, 0.92). Besides, the diagnostic criteria of CAL was not a dramatic impact factor, P=.080, t=-2.11, 95%CI (0.13, 1.16). Meanwhile, the meta-regression also did not detect the study design has a dramatic impact on the homogeneity of the enrolled studies too, P=.056, t=-2.37, 95%CI (0.07, 1.05). After then, we conducted two subgroups analysis by the study design and the total sample size. The pooled DOR of the prospective group was 32.52 (95% CI, 14.12 - 74.89) with low heterogeneity (P=.2094, Cochran-Q=4.53,	2-3

		I2=33.8%), and the calculated AUC value was 0.9065±0.0577. Besides, the pooled DOR of the retrospective group was 8.41 (95% CI, 5.19 - 13.62) with no significant heterogeneity (P=.4407, Cochran-Q=2.70, I2=0.0%), and the calculated AUC value was 0.7357±0.0692. Meanwhile, the pooled DOR of the total sample size (n ≤ 100) group was 18.37 (95% CI, 8.76 - 38.54) with no significant heterogeneity (P=.3729, Cochran-Q=3.12, I2=4%), and the calculated AUC value was 0.8064±0.1173. Additionally, the pooled DOR of the total sample size (n > 100) group was 11.73 (95% CI, 4.36 - 31.54) with moderate heterogeneity (P=.0184, Cochran-Q=10.02, I2=70.1%), and the calculated AUC value was 0.8976±0.0522.	
**DISCUSSION**			
Summary of evidence	24	The present study showed that the overall diagnostic sensitivity and specificity of NT-proBNP for diagnosis KD with CAL in the acute phase were 0.84 and 0.71, AUC of SROC was 0.8582. Then, we evaluated five studies, which all had thresholds at approximately 900 ng/L. Finally, we found the overall diagnostic sensitivity and specificity of NT-proBNP (threshold ≈ 900 ng/L) for diagnosis KD with CAL were 0.82 and 0.72, AUC of SROC was 0.8868, which was slightly higher than the overall diagnostic accuracy of NT-proBNP. In general, those found suggested that NT-proBNP may be used as a biomarker for detecting CAL of KD, and the recommended threshold was 900 ng/L.	4
Limitations	25	There are several limitations of this meta-analysis. First, the number of included studies was small (n = 8), and all of them were conducted in Asian populations, which means these results may not generalize to other areas. Second, no included articles combine NT-proBNP with other laboratory tests, such as ESR, CRP, to identify the diagnostic accuracy of KD with CAL, which could work as a better method for detection. Third, all of the follow-up time of the included articles were unclear, which may lead to the deviation of CAL diagnosis, which could further affect the accuracy of NT-proBNP in predicting CAL of KD.	4-5
Conclusions	26	In conclusion, despite these limitations, this is the first meta-analysis showed that NT-proBNP could be used as a biomarker for detecting CAL of KD, and the recommended threshold was 900 ng/L. Besides, further well-designed studies are needed to strictly evaluate the value of NT-proBNP in predicting CAL of KD.	5
**FUNDING**			
Funding	27	This work was supported by grants from the National Natural Science Foundation of China (No. 81700360, 81741025, 81570369 and 81571515) and the Technologic Program from Sichuan Province Government Foundation, China (No. 2018JY0603).	8-9

From: Moher D, Liberati A, Tetzlaff J, Altman DG, The PRISMA Group (2009). Preferred Reporting Items for Systematic Reviews and Meta-Analyses: The PRISMA Statement. PLoS Med 6(7): e1000097. doi:10.1371/journal.pmed1000097

For more information, visit: **www.prisma-statement.org**.

N-terminal pro-brain natriuretic peptide as a biomarker for predicting coronary artery lesion of Kawasaki disease

Xiaolan Zheng[1,2], Yi Zhang [1,2], Lei Liu[1,2], Peng Yue[1,2], Chuan Wang [1,2], Kaiyu Zhou[1,2], Yimin Hua[1,2], Gang Wu[1,2] & Yifei Li[1,2]

S Appendix 1. Search strategies for EMBASE, the Cochrane Central Register of Controlled Trials, and China National Knowledge Infrastructure.

A: Embase database was searched as follows: #1 'mucocutaneous lymph node syndrome' OR 'Kawasaki disease' OR ‘Kawasaki syndrome', #2 'pro-brain natriuretic peptide' OR 'NT-proBNP' OR 'N-terminal pro-BNP' OR 'NTproBNP' OR 'NT-BNP', #1 AND #2.

Embase		
1	('mucocutaneous lymph node syndrome' or 'Kawasaki disease' or 'Kawasaki syndrome').mp. [mp=title, abstract, heading word, drug trade name, original title, device manufacturer, drug manufacturer, device trade name, keyword, floating subheading word, candidate term word]	10019
2	('pro-brain natriuretic peptide' or 'NT-proBNP' or 'N-terminal pro-BNP' or 'NTproBNP' or 'NT-BNP').mp. [mp=title, abstract, heading word, drug trade name, original title, device manufacturer, drug manufacturer, device trade name, keyword, floating subheading word, candidate term word]	20018
3	#1 AND #2	123

B: the Cochrane Central Register of Controlled Trials database was searched as follows: (‘mucocutaneous lymph node syndrome’ OR ‘Kawasaki disease’ OR ‘Kawasaki syndrome’) AND (‘pro-brain natriuretic peptide’ OR ‘NT-proBNP’ OR ‘N-terminal pro-BNP’ OR ‘NTproBNP’ OR ‘NT-BNP’) in Title Abstract Keyword

the Cochrane Central Register of Controlled Trials		
1	(‘mucocutaneous lymph node syndrome’ OR ‘Kawasaki disease’ OR ‘Kawasaki syndrome’) in Title Abstract Keyword	233
2	(‘pro-brain natriuretic peptide’ OR ‘NT-proBNP’ OR ‘N-terminal pro-BNP’ OR ‘NTproBNP’ OR ‘NT-BNP’) in Title Abstract Keyword	1554
3	#1 AND #2	4

C: China National Knowledge Infrastructure database was searched as follows: (mucocutaneous lymph node syndrome OR Kawasaki disease OR Kawasaki syndrome) AND (pro-brain natriuretic peptide OR NT-proBNP OR N-terminal pro-BNP OR NTproBNP OR NT-BNP) in Title Abstract Keyword.

China National Knowledge Infrastructure database		
1	(mucocutaneous lymph node syndrome OR Kawasaki disease OR Kawasaki syndrome) in Title Abstract Keyword	7208
2	(pro-brain natriuretic peptide OR NT-proBNP OR N-terminal pro-BNP OR NTproBNP OR NT-BNP) in Title Abstract Keyword	17028
3	#1 AND #2	108
